# Structural Domains of the *Bacillus thuringiensis* Vip3Af Protein Unraveled by Tryptic Digestion of Alanine Mutants

**DOI:** 10.3390/toxins11060368

**Published:** 2019-06-21

**Authors:** Yudong Quan, Juan Ferré

**Affiliations:** ERI de Biotecnología y Biomedicina (BIOTECMED), Department of Genetics, Universitat de València, 46100 Burjassot, Spain; quan@uv.es

**Keywords:** Bt toxins, insecticidal proteins, trypsin cleavage, tetrameric proteins, domain map

## Abstract

Vip3 proteins are increasingly used in insect control in transgenic crops. To shed light on the structure of these proteins, we used the approach of the trypsin fragmentation of mutants altering the conformation of the Vip3Af protein. From an alanine scanning of Vip3Af, we selected mutants with an altered proteolytic pattern. Based on protease digestion patterns, their effect on oligomer formation, and theoretical cleavage sites, we generated a map of the Vip3Af protein with five domains which match some of the domains proposed independently by two in silico models. Domain I ranges amino acids (aa) 12–198, domain II aa199–313, domain III aa314–526, domain IV aa527–668, and domain V aa669–788. The effect of some mutations on the ability to form a tetrameric molecule revealed that domains I–II are required for tetramerization, while domain V is not. The involvement of domain IV in the tetramer formation is not clear. Some mutations distributed from near the end of domain I up to the end of domain II affect the stability of the first three domains of the protein and destroy the tetrameric form upon trypsin treatment. Because of the high sequence similarity among Vip3 proteins, we propose that our domain map can be extended to the Vip3 family of proteins.

## 1. Introduction

Vip3A proteins are produced during the vegetative phase of growth of *Bacillus thuringiensis* and are of practical interest because of their insecticidal activity against Lepidoptera [1]. Because Vip3A proteins share no sequence and structural homology with *B. thuringiensis* Cry proteins, they are considered an excellent complement to Cry proteins in crop protection and resistance management. Some commercial Bt-crops (crops protected from insect attacks by expressing insecticidal proteins from *B. thuringiensis*) combine Cry and Vip3 proteins, and this strategy of pyramiding proteins with different modes of action is expected to continue in the future [2].

Despite the increasing interest in Vip3 proteins, their mode of action is not completely understood, and their 3D structure still remains unknown. Recently, a number of studies have provided valuable information toward the structure of these proteins. Multiple-sequence alignments of Vip3 proteins have shown that they contain between 786 and 803 amino acids (corresponding to a molecular weight of around 89 kDa), with a highly conserved N-terminal part (up to residue 334) and a highly variable C-terminal region [1]. Proteolytical activation in the midgut of insects eliminates a small part of the N-terminus, which, in the case of Vip3A, takes place at residue R11/12 [3,4] and, in the case of Vip3Bc1, at R20 [3], followed by the cleavage of the protein at the primary cleavage site, which in Vip3Aa and Vip3Af is K198/D199 [4,5]. Then, two peptides, of about 19 and 65 kDa, are generated, and these remain strongly bound to each other [3,6,7]. More recently, it has been shown that Vip3 proteins are found in solution as homo-tetramers, both as protoxins and after activation by proteases [3,7,8,9].

To date, a high resolution 3D structure of a Vip3 tetrameric protein is lacking, though low resolution images have been obtained [8,10]. In an attempt to propose a 3D structure for Vip3 proteins, Vip3Af1 and Vip3Aa16 have been subjected to in silico modelling, and several domains have been proposed. For Vip3Af, five structural domains were proposed [4], with domain 1 spanning from the N-terminus to residue 188, domain 2 from residue 189 to 272, domain 3 from 273 to 542, domain 4 from 543 to 715, and domain 5 from 716 to the end. For Vip3Aa16, three domains were proposed, though domain 1 was further subdivided into three domains [11]: Subdomain 1.1 spanned from the N-terminus to residue 313, subdomain 1.2.1 from 314 to 441, subdomain 1.2.2 from 442 to 532, domain 2 from 533 to 667, and domain 3 from 668 to the end. Given the high sequence similarity between the two proteins (92.7%), the discrepancy between them regarding the regions spanned by the domains just reflects inaccuracies of the modelling programs used, probably due to the low availability of reference sequences with known 3D structures.

With the aim of shedding light on the putative functional and structural domains of Vip3 proteins, we have made use of selected Vip3Af alanine mutants (Ala-mutants) from a previous work [4] which drastically affect insecticidal activity. Most of these mutants are distributed in three clusters along the length of the protein and show altered proteolytic patterns upon trypsin digestion [4]. Banyuls et al. labeled these altered patterns as “a” to “f” [4]. In the present work, we have made use of these critical Ala-mutants with the rationale that the altered patterns, generated by conformational changes due to the residue substitution, may unravel structural and functional domains. The results, based on protease digestion patterns, oligomer formation, and theoretical tryptic sites, have allowed us to propose a map of the Vip3Af protein with five domains. The information thus generated will contribute to the better understanding of the structure of Vip3 proteins and may be useful in the search of the 3D structure of this family of proteins.

## 2. Results

### 2.1. Effect of Residue Substitution on the Proteolytic Cleavage of Vip3Af

Changes in protein conformation may expose potential cleavage sites otherwise buried inside the protein which, when exposed to proteases, give rise to altered patterns of fragments compared with that of the wild type protein (WT). Proteolytic patterns may thus unravel structural domains in the Vip3Af protein.

**Figure 1:**
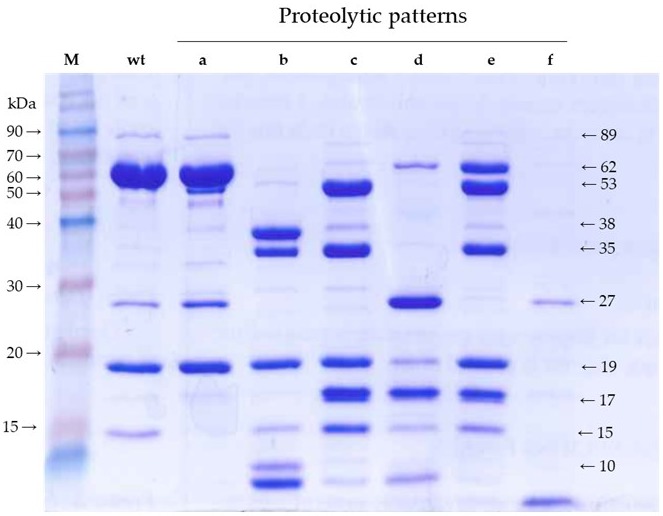
Trypsin digestion of Vip3Af(WT) and some of the selected mutants (one representative of each of the patterns “a” to “f”) after SDS-PAGE. The proteins were treated with 5% trypsin (*w*/*w*) at 30 °C for 24 h and then stopped with the addition of an irreversible trypsin protease inhibitor (1 mM 4-(2-aminoethyl)benzenesulfonyl fluoride (AEBSF)) at room temperature for 10 min). M: Molecular weight markers. Patterns “a” to “f” were obtained from mutants T167A, W552A, I699A, F229A, G689A, and E483A, respectively.

We confirmed the altered proteolytic patterns obtained before with Ala-mutants [4]. To better define the major fragments generated by the action of trypsin on each of the mutants, we used an irreversible trypsin inhibitor to terminate the reaction and avoid further processing during SDS denaturation before gel loading [5,7]. Figure 1 shows the SDS-PAGE separation of the tryptic fragments from six selected mutants (T167A, F229A, E483A, W552A, G689A, and I699A). Regarding the major fragments, Vip3Af(WT) and mutant T167A showed the 65 and 19 kDa bands (pattern “a”) as a result of the cleavage at the primary cleavage site after residue K198. The rest of the patterns lacked the 65 kDa band. Patterns “b”, “c”, and “e” contained the 19 kDa band, indicating that they altered the C-terminal part of the protein but not the N-terminal part. Patterns “d” and “f” did not contain the 19 kDa band either, indicating that the conformational change had a larger effect on the overall structure of the protein. Pattern “b” and “c” share the 35 and 19 kDa bands in common; in addition, the former showed strong bands of 38 and 10 kDa, whereas the latter showed main bands of 53, 17 (a doublet), and 15 kDa. Patterns “d” and “f” lack large fragments (larger than30 kDa); instead, they share a main band of 27 kDa; in addition, pattern “d” has a strong band of 17 kDa, and pattern “f” has a strong band of <10 kDa. Finally, pattern “e” is the same as pattern “c” but still maintains the band of 65 kDa, suggesting that mutant G689A (the only representative of pattern “e”) was relatively stable compared with those mutants with pattern “c”.

### 2.2. Insecticidal Activity of the Ala-Mutants after Trypsin Treatment

The mutants selected in this study had been shown to have decreased insecticidal activity when tested as protoxins [4]. Here we tested the activity of the most drastic ones after in vitro treatment with trypsin (Table 1). The results were similar to the ones reported previously for the protoxin form, confirming that these mutations have a strong deleterious effect on the insecticidal activity of the protein. It is worth mentioning the differences in insecticidal activity observed among mutants with the same proteolytic pattern—between P171A and F229A (both giving rise to pattern “d”), and among I699A, Y719A, and G727A, for example (all giving rise to pattern “c”). This might be explained by either the effect of the residue substitution on intra- or intermolecular interactions or by differences in their stability to proteases. Mutant G689A, which gives pattern “e” after trypsin treatment, is the most toxic one among those tested. As mentioned above, this mutant has the same band pattern as pattern “c” with an extra 65 kDa band, reflecting its higher stability compared with mutants giving pattern “c”.

### 2.3. Effect of Residue Substitution on Vip3Af Oligomerization

**Figure 2:**
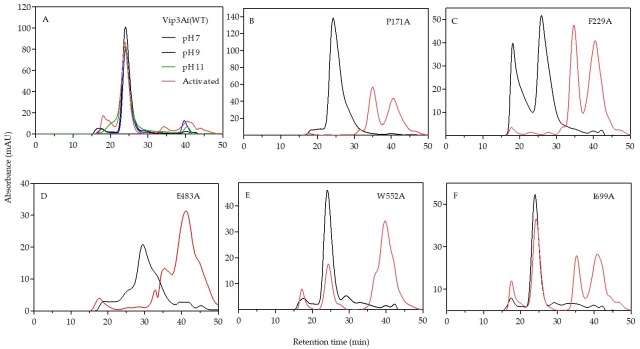
Gel filtration chromatography of Vip3Af(WT) and representative Ala-mutants. A Tris buffer (50 mM Tris, 150 mM NaCl, pH 9.0) was used in all cases (black line: Protoxin; red line: Trypsin-treated). For the Vip3Af(WT), elution was also performed in a phosphate buffer (50 mM phosphate, 150 mM NaCl, pH 7.0) (blue line) and a carbonate buffer (50 mM Na_2_CO_3_, 150 mM NaCl, pH 11.0) (green line). (**A**) Vip3Af1(WT); (**B**) P171A; (**C**) F229A; (**D**) E483A; (**E**) W552A; (**F**) I699A.

**Figure 3:**
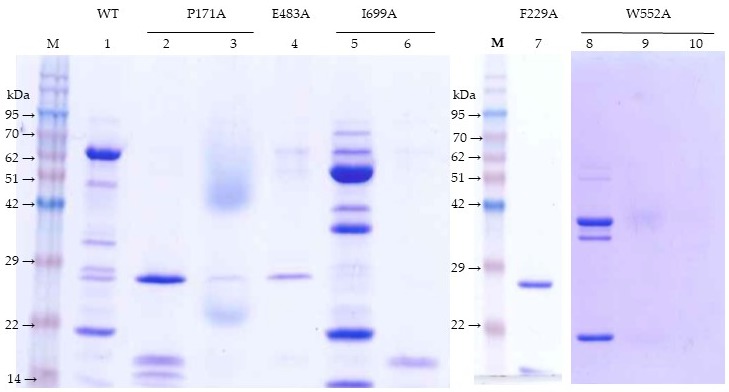
SDS-PAGE analysis of the chromatographic peaks of the trypsin-activated mutants from Figure 2. Lane 1, Vip3Af(WT) peak at 24–26 min; lane 2, P171A peak at 34–36 min; lane 3, P171A peak at 40–42 min; lane 4, E483A peak at 34–36 min; lane 5, I699A peak at 24–26 min; lane 6, I699A peak at 34–36 min; lane 7, F229A peak at 34–36 min; lane 8, W552A peak at 24–26 min; lane 9, W552A peak at 38–40 min; lane 10, W552A peak at 40–42 min.

Residue substitutions may affect the capacity of the Vip3Af protein to form the tetramer [7], the form that Vip3 proteins adopt in solution [3,7,8,9,10]. We used gel filtration chromatography to determine the oligomerization state of the Ala-mutants, both as protoxins and after trypsin treatment. First, we tested the wild type Vip3Af (from now on: Vip3Af(WT)) and determined the possible effect of pH on oligomerization. Figure 2A shows that, at the pH range tested (pH 7, 9, and 11), there was no effect on the tetramerization of the Vip3Af(WT) protoxin. The chromatograms showed a main peak at 24 min, corresponding to a molecular weight of approximately 370 kDa (a tetramer of the 89 kDa protoxin should theoretically be of 356 kDa). The trypsin-treated Vip3Af(WT) also showed just one peak at 24 min (Figure 2A). An SDS-PAGE analysis of the peak showed the 19 and 65 kDa bands (Figure 3, lane 1), confirming that trypsin treatment did not induce the separation of the two fragments [3,5,6,7,9]. Mutants T167A and E168A (both giving rise to pattern “a”) showed chromatograms that did not differ from that of the wild type (not shown).

With mutant P171A (which gives rise to pattern “d”), the protoxin eluted at 24 min, revealing a tetrameric form; however, just small fragments (eluting at 34.8 min) were observed after trypsinization (Figure 2B). SDS-PAGE of the peak at 34.8 min showed a 27 kDa strong band (Figure 3, lane 2). This chromatography profile was also observed in mutants L209A and M238A (both giving rise to pattern “d”) (not shown). The chromatogram of the F229A protoxin showed big peaks at 18 and 26 min (Figure 2C), the former coinciding with the exclusion volume of the column and corresponding to protein aggregates. The peak at 26 min indicated a molecular weight of approximately 230 kDa, which would best fit a dimeric form of the protein. This mutant (which renders pattern “d”) also showed only small fragments eluting at 34.8 min after trypsinization. SDS-PAGE of the fraction at 34.8 min revealed a main fragment of 27 kDa (Figure 3, lane 7).

Neither the protoxin nor the trypsin-treated mutant E483A (which gives pattern “f”) showed any tetramer in solution (Figure 2D); the main peak of the protoxin eluted at 29.5 min, corresponding to an approximate estimated molecular weight of 122 kDa (best fitting a monomer), whereas the trypsinized protein eluted at around 35 min, corresponding to the 27 kDa fragment and smaller fragment (<10 kDa), as revealed by SDS-PAGE (Figure 3, lane 4).

Mutant W552A (which gives rise to pattern “b”) formed a tetramer both as a protoxin and after trypsin treatment (Figure 2E); however, upon trypsin treatment, the amount of tetramer was reduced, and a large peak corresponding to small fragments appeared. An analysis of the fraction at 24 min by SDS-PAGE showed the presence of the 38, 35, and 19 kDa fragments (Figure 3, lane 8).

Mutant I699A (which renders pattern “c”) also tetramerized as a protoxin and after trypsin treatment (Figure 2F); however, after trypsinization, it also showed fragments eluting at 35.2 min. This chromatography profile was also observed in the rest of mutants which give rise to pattern “c” (Y719A and G727A) and “e” (G689A). The SDS-PAGE analysis of the fraction at 24 min revealed, among other minor bands, the 53, 35, and 19 kDa fragments (Figure 3, lane 5), whereas the fraction at 35.2 min revealed a fragment of 17 kDa (Figure 3, lane 6).

From the chromatographic analysis, we can conclude that the 27 and 17 kDa fragments, once cleaved by trypsin, are released from the structure and no longer form part of the oligomer. However, the tetrameric structure of Vip3Af can still be maintained in the presence of fragments 38, 35, and 19 kDa (such as in mutant W552A, which gives pattern “b”) or fragments 53, 35, and 19 kDa (such as mutants rendering pattern “c”). Residues F229 and E483 must have a key role in the oligomerization of the Vip3Af protoxin, since their exchange for alanine prevents tetramer formation even before trypsin treatment.

### 2.4. Identification of 17, 27, and 38 kDa Tryptic Fragments by Peptide Fingerprinting 

The identification of the tryptic fragments was performed after the separation of the fragments by 2D gel electrophoresis and/or size filtration chromatography followed by SDS-PAGE. The results of the peptide fingerprint were matched with those of the tryptic sites in the primary sequence of Vip3Af, and the estimated size of the fragment was taken into account to set the fragment limits. Through 2D gel electrophoresis, we could separate and analyze the 17 and 27 kDa spots from the trypsin-treated F229A mutant. The results of the peptide fingerprint, along with the tryptic sites in the sequence of Vip3Af, indicated that the 27 kDa fragment corresponded to residues from 523/526 to probably the end of the protein. The same type of analysis with the spot of 17 kDa indicated that it corresponded to residues from 523/526 to 661/663. The 17 kDa fragment from the trypsin-treated I699A mutant was analyzed from the chromatographic fraction B15 (peak 34.8 min) of this mutant. The results indicated the same match as the 17 kDa fragment from mutant F229A. The identity of the 38 kDa fragment was determined from the chromatography fraction A11 (24.2 min) from trypsin-treated W552A; the peptide fingerprint indicated that the fragment corresponded to residues from 313/315 to 661/668.

## 3. Discussion

Banyuls et al. [4] defined six proteolytic patterns of mutants with strongly impaired insecticidal activity. With minor modifications in the methodology, we have confirmed and refined such patterns with the aim of revealing the major fragments generated by trypsin and then identifying their position in the primary structure of the protein. The only difference observed with the previous proteolytic patterns is that, using the irreversible trypsin inhibitor to stop the reaction, we obtained a strong band of 35 kDa in patterns “b” and “c”, which was not observed before. We also detected bands smaller than 19 kDa by stopping the electrophoresis before they ran out of gel. Altogether, we ended up with fragments of 53, 38, 35, 27, 19, 17, 15, and <10 kDa, most of them shared by various patterns. We hypothesized that the limits of these fragments may correlate with the structural domains of the wild type protein.

**Figure 4:**
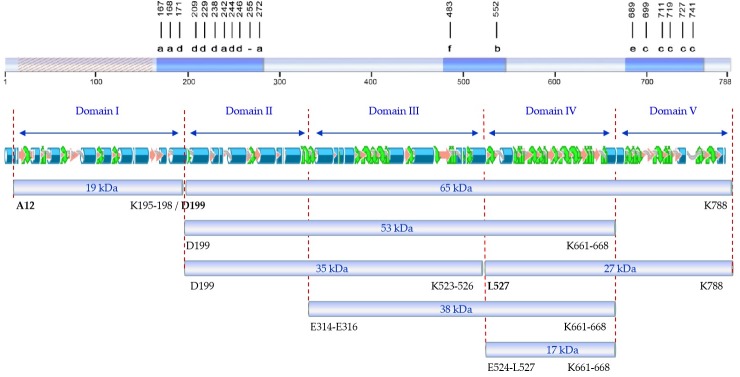
Schematic representation of the Vip3Af protein. (**a**) Distribution of critical residues affecting the insecticidal activity (dashed region: Non-analyzed), with indication of the band pattern after trypsin treatment, following Banyuls et al. [4]; (**b**) proposed structural domains as defined by the tryptic fragments; (**c**) predicted secondary structure of Vip3Af, following Banyuls et al. [4]; (**d**) main fragments after trypsin treatment of the Vip3Af protein and their Ala-mutants (amino acid residues identified by Edman’s degradation are shown in bold).

In a previous study, Banyuls et al. [4] identified the tryptic fragments of 62 (here referred to as 65), 55 (here referred to as 53), 27, and 20 (here referred to as 19) kDa. Our peptide fingerprint results of fragments of 17, 27, and 38 kDa, taking into account the tryptic sites in the sequence of Vip3Af, allowed us to define their position in the sequence of the protein. Putting all this information together, we propose a map of the tryptic fragments such as the one shown in Figure 4, which defines five domains. In this map, domain I spans the region covered by the 19 kDa fragment (from residues 12 to 198); domain II spans the region from the primary cleavage site to the start of the 38 kDa fragment (from residues 200 to 313/315); domain III spans from the start of the 38 kDa fragment up to the start of fragments of 17 and 27 kDa (from residues E314-E316 to 523/526); domain IV spans the 17 kDa fragment (from residue 524/527 to residue 661/668) and basically consists of the carbohydrate-binding motif common to all Vip3 proteins with the exception of Vip3Ba [1]; and domain V spans from the end of the 17 kDa fragment (and also the end of the 38 and 53 kDa fragments) to the end of the protein (from residue 662/669 to 788). Compared with the proposed domains by in silico modelling, the domain I proposed by us is in good agreement with domain 1 proposed by Banyuls et al. [4] for Vip3Af (from 1 to 188), though there is no further correlation between both models for the rest of domains. However, the boundaries between domain II and III, III and IV, and IV and V in our proposed map have their correspondence with the domains proposed by Sellami et al. [11] for Vip3Aa (at residues 313, 532, and 667, respectively). The agreement between the domain limits proposed by us with some of those defined by in silico modelling supports the predictive value of the tryptic fragments approach to unravel structural domains of the Vip3A proteins.

The results from gel filtration chromatography of the Ala-mutants shed light on the structural role of the proposed domains. Mutants rendering patterns “b”, “c”, and “e” are found forming a tetramer both as protoxins and also after trypsin treatment. Since trypsin digests the 27 kDa fragment (the only one containing domain V), we can conclude that domain V is not necessary to maintain the oligomeric structure. All these mutants, after trypsin treatment, have in common fragments of 19 kDa (domain I) and 35 kDa (domains II and III), plus another larger fragment (either of 38 or 53 kDa) which includes domain IV. Despite the fact that the 17 kDa fragment (which corresponds to domain IV) elutes separately from the tetramer in the chromatography of mutants with patterns “c” and “e”, the tetramer contains domain IV in the structure as part of the 53 kDa fragment. Therefore, according to the results, domains I–III are required to form the tetrameric structure, the need for domain IV is not clear, and domain V is not necessary.

An interesting observation from patterns “b” and “c” is that, in addition to the 19 kDa band, the sum of the remaining main bands gives an apparent molecular weight exceeding that of 65 kDa. In the case of pattern “b”, these bands correspond to fragments of 35 and 38 kDa. In the case of pattern “c”, the strongest bands are those corresponding to fragments of 15, 17, 19, 35, and 53 kDa. Therefore, there must be an alternative splicing of the 65 kDa fragment in mutants rendering these two patterns.

The requirement of domain I to form the tetramer, along with domain exchange studies between the 19 kDa fragment and the rest of the protein with Vip3Ab and Vip3Bb [3], support the functional role of this domain and rules out the early beliefs that the 19 kDa fragment was non-essential in the insecticidal activity of Vip3 proteins and that only the 65 kDa fragment was the active core. It has been reported that complete deletion of the first 198 N-terminal amino acids in Vip3Aa completely abolishes its toxicity and produces a 62 kDa protein highly sensitive to trypsin degradation [12]. However, some studies have shown that domain I can withstand short N-terminal deletions without affecting the insecticidal activity [13,14]. In contradiction to the above results, Gayen et al. [15] reported an active Vip3Aa protein without domain I.

From the distribution of Ala-mutants with decreased insecticidal activity in the primary structure of Vip3Af (Figure 4), we can observe that they gather into two clusters, except for mutant E483A (the only representative of pattern “f”) and mutant W552A (the only representative of pattern “b”). The first cluster contains all mutants with either pattern “a” or “d”. Mutations altering the structure and giving pattern “d” are concentrated at the end of domain I and the first part of domain II. This region of the protein, around the primary cleavage site, must have an important role in maintaining the 19 and 65 kDa fragments together, and this might be essential to preserve the overall structure of the tetrameric protein. The second cluster is in domain V and contains all the mutants with either pattern “c” or “e”. These mutants destabilize domain V, which is further digested by trypsin with the result of fragment 27 kDa being converted to the 17 kDa fragment.

## 4. Conclusions

Using the approach of the trypsin fragmentation of mutants altering the conformation of the Vip3Af protein, we have defined five domains in the structure of Vip3Af which match some of the domains proposed independently by two in silico models. The effect of some the mutations on the ability to form a tetrameric molecule reveals that domains I–III are required for tetramerization, while domain V is not. The involvement of domain IV in the tetramer formation is not clear. The overlapping fragments in the proteolytic patterns suggest a tetramer with a distinct disposition of the monomers, in such a way that the tryptic sites exposed in two molecules are different to those exposed in the other two. Residues around the primary cleavage site are important for maintaining the structure of the protein, since trypsin processing in mutants of pattern “d” digests most part of the protein and destroys the tetrameric form. Mutants in domain V belonging to pattern “c” destabilize this domain, though they not affect the tetrameric structure after trypsin processing. Because of the high sequence similarity among Vip3 proteins, we think that our domain map proposal may be valid for the Vip3 family of proteins. The information provided here may help to further clarify the 3D structure and its implications in the mode of action of Vip3 proteins.

## 5. Materials and Methods

### 5.1. Protein Source, Expression and Purification

The source of the 788 amino acid protein Vip3Af1(WT) (NCBI accession No. CAI43275) and that of its mutant proteins has been described in Banyuls et al. [4]. The mutant proteins, all with decreased insecticidal activity, differed from Vip3Af(WT) and from each other, by a single amino acid residue which had been changed to an alanine residue. For this work, we selected the mutants which decreased the toxicity: T167A, E168A, P171A, L209A, F229A, M238A, E483A, W552A, G689A, I699A, Y719, and G727. The expression and purification of Vip3Af(WT) and the mutant proteins was carried out as described before [4], using 1 mL HisTrap FF columns (GE Healthcare Bio-Sciences AB, Uppsala, Sweden). Vip3Af proteins were eluted with a phosphate buffer (50 mM phosphate, 300 mM NaCl, pH 7.4) containing 150 mM imidazole, and 1 mL fractions were collected in tubes containing 50 µL of 0.1 M ethylenediaminetetraacetic acid (EDTA). Fractions with a high protein concentration (determined photometrically at 280 nm) were pooled and dialyzed overnight at 4 °C against a TNE buffer (20 mM Tris-HCl, 150 mM NaCl, 5 mM EDTA, pH 8.6). The purity of the preparation (10 µL) was checked by SDS-PAGE, and the protein concentration was determined by the Bradford’s method. After dialysis, the proteins were stored at −20 °C until used.

### 5.2. Trypsin Treatment and SDS-PAGE Analysis of the Tryptic Fragments

The purified Vip3Af protoxins were subjected to proteolytic activation with commercial trypsin (trypsin from bovine pancreas, SIGMA T8003, Sigma-Aldrich, St. Louis, MO, USA). A mixture of protein:trypsin (5:100, *w*/*w*), in a TNE buffer was incubated at 30 °C for 24 h. Aliquots (10 µL ) of the trypsinized proteins were subjected to 12% SDS-PAGE. Prior to electrophoresis, the samples were made 1 mM with an 4-(2-aminoethyl)benzenesulfonyl fluoride (AEBSF) protease inhibitor (ThermoFisher, Waltham, MA, USA), left standing for 10 min at room temperature, and then heated at 100 °C for 5 min with a loading buffer (0.2 M Tris-HCl pH 6.8, 1 M sucrose, 5 mM EDTA, 0.1% bromophenol blue, 2.5% SDS, and 5% β-mercaptoethanol) (2:1, sample:loading buffer). The trypsin-treated samples to be used for chromatography and bioassays were stored at −20 °C for less than one week.

### 5.3. Insect Rearing and Bioassays

Insect rearing and bioassays were carried out in a rearing chamber maintained at 25 ± 2 °C, 70 ± 5% relative humidity, and 16:8 h light:dark on a semi-synthetic diet based on corn flour and wheat germ that contained yeast, ascorbic acid, and nipagin. Surface contamination assays were performed with 50 μL of protein sample on 2 cm^2^ diameter well plates. The concentration of Vip3Af protein was 1 µg/cm^2^, a concentration at which the Vip3Af(WT) kills 100% of the larvae. A Tris buffer (20 mM Tris-HCl, 150 mM NaCl, pH 8.6) was used as a blank control. Once the surface was dry, a neonate *S. frugiperda* larvae was gently placed into the well and then sealed. The number of dead and 1-instar larvae were recorded after 7 days. A larva was considered dead if it did not respond to mechanical stimulation. The mean mortality and functional mortality (dead larvae plus larvae that had not developed beyond the first instar) were determined from two replicates of 32 insects each.

### 5.4. Gel Filtration Chromatography

Gel filtration chromatography was performed with an ÄKTA explorer 100 chromatography system in a Superdex 200 10/300 GL column (GE Healthcare Life Sciences, Uppsala, Sweden) at a flow rate of 0.5 mL/min of a Tris buffer (50 mM Tris-HCl, 150 mM NaCl, pH 9.0), unless otherwise indicated. To estimate the molecular weight of the peaks, the column was calibrated with the following mix of standards: 4 mg/mL ovalbumin (44 kDa), 3 mg/mL conalbumin (75 kDa), 4 mg/mL aldolase (158 kDa), 0.3 mg/mL ferritin (440 kDa), 5 mg/mL thyroglobulin (6690 kDa), and Blue Dextran 200 (exclusion limit), dissolved in water.

### 5.5. Identification of Tryptic Fragments

Major bands (27 and 17 kDa) from the trypsin-treated F229A mutant were identified after separation in a 2D-gel. The 17 kDa band from the trypsin-treated I699A mutant was first separated by chromatography in the Superdex 200 column and then by SDS-PAGE. The 38 kDa band from the trypsin-treated W552A mutant was first isolated by Superdex 200 chromatography and then by SDS-PAGE. 

For the peptide identification, protein bands were directly cut out from the gel and digested with trypsin. The peptide mass and sequence were determined by liquid chromatography and tandem mass spectrometry (LC-MS/MS) in a nanoESI qQTOF (5600 TripleTOF, ABSCIEX, Framingham, MA, USA). The mass transitions were scanned first from 350–1250 m/z and then followed by a second scan from 100–1500 m/z. The peptides sequence identified were compared to the Vip3Af1(WT) protein sequence to match the region corresponding to each SDS-PAGE proteolytic band. Expected molecular weights were calculated using the online SIB Compute pI/Mw tool (https://web.expasy.org/compute_pi).

## Figures and Tables

**Table 1:** Toxicity, against *S. frugiperda*, of trypsin-treated Vip3Af and selected mutants, with indication of the proteolytic pattern after trypsin digestion. ^1^

Toxins	Tryptic Pattern	Mortality (%)	FM (%)
WT	a	100	100
T167A	a	13 ± 9	19 ± 11
E168A	a	0	3.1 ± 3.3
P171A	d	16.7 ± 4.7	40.0 ± 9.5
F229A	d	6.3 ± 7.2	6.3 ± 7.2
E483A	f	3.3 ± 3.6	16.7 ± 4.7
W552A	b	0	0
G689A	e	42 ± 30	48 ± 21
I699A	c	0	0
Y719A	c	26.7 ± 9.4	46.7 ± 0
G727A	c	0	0

^1^ Percent mortality and functional mortality (FM, defined as dead larvae plus larvae remaining at 1-instar) at 1 µg/cm^2^. Mean ± SD from two replicates of 32 insects each.

## References

[1] Chakroun M., Banyuls N., Bel Y., Escriche B., Ferré J. (2016). Bacterial vegetative insecticidal proteins (Vip) from entomopathogenic bacteria. Microbiol. Mol. Biol. Rev..

[2] Carriere Y., Fabrick J.A., Tabashnik B.E. (2016). Can pryamids and seed mixtures dely resistance to Bt Crops?. Trends Biotechnol..

[3] Zack M.D., Sopko M.S., Fery M.L., Wang X., Tan S.Y., Arruda J.M., Letherer T.T., Narva K.E. (2017). Functional characterization of Vip3Ab1 and Vip3Bc1: Two novel insecticidal proteins with differential activity against lepidopteran pests. Sci. Rep..

[4] Banyuls N., Hernández-Rodríguez C.S., Van Rie J., Ferré J. (2018). Critical amino acids for the insecticidal activity of Vip3Af from *Bacillus thuringiensis*: Inference on structural aspects. Sci. Rep..

[5] Bel Y., Banyuls N., Chakroun M., Escriche B., Ferré J. (2017). Insights into the structure of the Vip3Aa insecticidal protein by protease digestion analysis. Toxins.

[6] Chakroun M., Ferré J. (2014). *In vivo* and *in vitro* binding of Vip3Aa to *Spodoptera frugiperda* midgut and characterization of binding sites by ^125^I radiolabeling. Appl. Environ. Microbiol..

[7] Banyuls N., Hernández-Martínez P., Quan Y., Ferré J. (2018). Artefactual band patterns by SDS-PAGE of the Vip3Af protein in the presence of proteases mask the extremaly high stability of this protein. Int. J. Biol. Macromol..

[8] Palma L., Scott D., Harris G., Din S.U., Williams T., Roberts O., Young M., Caballero P., Berry C. (2017). The Vip3Ag4 Insecticidal protoxin from *Bacillus thuringiensis* adopts a tetrameric configuration that is maintained on proteolysis. Toxins.

[9] Şahin B., Gomis-Cebolla J., Güneş H., Ferré J. (2018). Characterization of *Bacillus thuringiensis* isolates by their insecticidal activity and their production of Cry and Vip3 proteins. PLoS ONE.

[10] Kunthic T., Surya W., Promdonkoy B., Torres J., Boonserm P. (2017). Conditions for homogeneous preparation of stable monomeric and oligomeric forms of activated Vip3A toxin from *Bacillus thuringiensis*. Eur. Biophys. J..

[11] Sellami S., Jemli S., Abdelmalek N., Cherif M., Abdelkefi-Mesrati L., Tounsi S., Jamoussi K. (2018). A novel Vip3Aa16-Cry1Ac chimera toxin: Enhancement of toxicity against *Ephestia kuehniella*, structural study and molecular docking. Int. J. Biol. Macromol..

[12] Li C., Xua N., Huanga X., Wanga W., Chenga J., Wu K., Shen Z. (2007). *Bacillus thuringiensis* Vip3 mutant proteins: Insecticidal activity and trypsin sensitivity. Biocontrol Sci. Technol. Biochem..

[13] Bhalla R., Dalal M., Panguluri S.K., Jagadish B., Mandaokar A.D., Singh A.K., Kumar P.A. (2005). Isolation, characterization and expression of a novel vegetative insecticidal protein gene of *Bacillus thuringiensis*. FEMS Microbiol. Lett..

[14] Selvapandiyan A., Arora N., Rajagopal R., Jalali S.K., Venkatesan T., Singh S.P., Bhatnagar R.K. (2001). Toxicity analysis of N- and C-terminus-deleted vegetative insecticidal protein from *Bacillus thuringiensis*. Appl. Environ. Microbiol..

[15] Gayen S., Hossain M.A., Sen S.K. (2012). Identification of the bioactive core component of the insecticidal Vip3A toxin peptide of *Bacillus thuringiensis*. J. Plant Biochem. Biotechnol..

